# Primary CALVARIAL tuberculosis: A case report

**DOI:** 10.1016/j.ijscr.2025.111084

**Published:** 2025-02-25

**Authors:** Abdulsemed Mohammed Yasin, Eyob Zenebe, Kibruyisfaw Zewude, Dagnachew Tamrat Belete, Betelhem Gebreamlak

**Affiliations:** Department of Surgery, Neurosurgery Unit, School of Medicine, Addis Ababa University, Ethiopia

**Keywords:** Calvarium, Tuberculosis, Tuberculoma, Chemotherapy, Extrapulmonary tuberculosis

## Abstract

**Introduction:**

Calvarial tuberculosis is a rare manifestation of extra pulmonary tuberculosis. Primary calvarial tuberculosis, with no evidence of tuberculosis elsewhere in the body, is an even rarer entity. Most cases are often misdiagnosed as osteomyelitis, syphilis, or bony metastasis among others.

**Case Presentation:**

We report a case of primary calvarial tuberculosis in a 17 years old female with complaints of progressively increasing swelling over the right frontoparietal region and headache with no history of previous tuberculosis. The patient was operated, and histopathological examination of excised tissue was suggestive of tubercular pathology. The patient is doing well after surgery and anti-tubercular therapy.

**Discussion:**

Skeletal tuberculosis occurs in approximately 1 % of cases of mycobacterial infection, and calvarial tuberculosis accounts for 0.2 % to 1.3 % of all cases of skeletal tuberculosis. Early clinical signs are usually absent. The emergence of a painless, fluctuant swelling stands out as the most common presentation. Radiological findings, which are very valuable tools to reach a diagnosis, are known to be variable and nonspecific. The gold standard for diagnosis is the demonstration of Acid Fast Bacilli (AFB) on microscopy and growth on culture. Treatment includes surgery and antituberculous therapy.

**Conclusion:**

Primary calvarial tuberculosis is a rare entity that can present with diverse symptoms and mimic other pathologies. A high index of suspicion should be maintained, especially in endemic areas, to ensure timely and accurate diagnosis. Surgery and antituberculous therapy remain the treatment of choice. Follow up is crucial to monitor for recurrence and other associated complications.

## Highlights

• **Tuberculosis (TB)** poses a substantial public health challenge, particularly in developing nations
• Involvement of the Calvarium in tubercular disease is rare, and even rarer is primary calvarial tuberculosis
• The diagnosis of primary calvarial tuberculosis is challenging and it requires the use of different diagnostic methodologies
• The gold standard for diagnosis calvarial tuberculosis is the demonstration of AFB on microscopy and growth on culture
• CT scan and MRI is very important in choosing the mode of treatment which usually include chemotherapy (antituberculous) and surgery.

## 1 Introduction

Tuberculosis (TB) poses a substantial public health challenge, particularly in developing nations. Factors such as malnutrition, overcrowding, inadequate sanitation, poor hygiene practices, lack of awareness, and delays in seeking medical attention significantly contribute to the transmission and impact of this disease. [1]

Calvarial tuberculosis, although rare, represents an unusual manifestation of a common disease and it occurs in only 0.2 % to 1.3 % of all cases of skeletal tuberculosis. [2] Skeletal tuberculosis, in general, is observed in approximately 1 % of cases of mycobacterium infection.

It was first reported by Reid in 1842 and the term primary calvarial tuberculosis is used when no evidence of tuberculosis is detected elsewhere in the body. [3] Involvement of the calvarium in tubercular disease is rare, and even rarer is primary calvarial tuberculosis, with no evidence of tuberculosis elsewhere in the body. [4]

Skeletal tuberculosis arises from the hematogenous spread of tubercle bacilli into the marrow spaces. The infrequency of calvarial tuberculosis can be attributed to the scarcity of cancellous (spongy) tissue in the flat bones of the skull and the absence of lymphatic vessels within them. [3] When calvarial TB does occur, it tends to affect the frontal and parietal bones, which have a greater amount of diploic space.

The common constitutional symptoms are absent and patients usually present with painless, fluctuant swelling over the scalp. [2] Headache If present, headache is usually localized to the site of the swelling. [4] Depending on the intracranial extension of the lesion, patients may rarely present with seizures or motor deficits. [4]

The diagnosis of primary calvarial tuberculosis is challenging and it requires the use of different diagnostic methodologies. The Mantoux test and raised erythrocyte sedimentation rate (ESR) are good markers and may provide diagnostic clues to TB. [5] But the gold standard for diagnosis is the demonstration of AFB on microscopy and growth on culture. [3] Radiological findings, which are very valuable tools to reach a diagnosis, are known to be variable and nonspecific. [6] Computed tomography (CT) scan and magnetic resonance imaging (MRI) are very important in choosing the mode of treatment, which usually includes chemotherapy and surgery.

This case is reported on accordance with SCARE criteria [7].

## 2 CASE

A 17-year-old school girl came to our Neurosurgery Outpatient Department (OPD) with progressively increasing swelling over the right frontoparietal region of 6 months duration. She also had a headache localized to the site of the swelling. Otherwise, she had no constitutional symptoms of tuberculosis and other neurologic complaints such as seizures or weakness. She had no personal or family history of tuberculosis.

She had a history of a fall accident 1 year and 6 months ago, which resulted in scalp swelling, a bruise, and a depressed skull fracture (DSF) in the right frontal area (the same site as the current swelling), managed conservatively. There was fluctuant swelling over the right frontoparietal area measuring 10 × 7 cm with a palpable bone defect. Except for tenderness, there were no signs of inflammation or skin color changes. There were multiple palpable bone defects in the right frontal area. General physical and routine hematological examination did not reveal any abnormality.

**Fig. 1:**
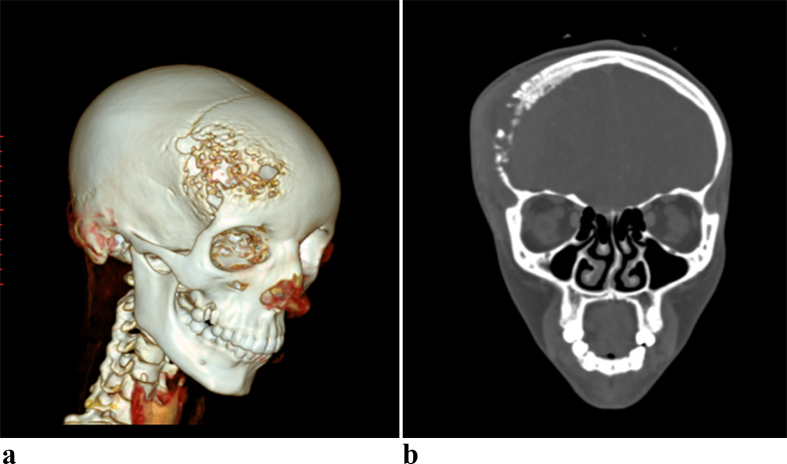
a. 3D reconstruct of Computed tomography showing multiple bony defect of the right frontal bone b. Bone window of Computed tomography showing right frontal bone destruction with sub galeal swelling.

**Fig. 2:**
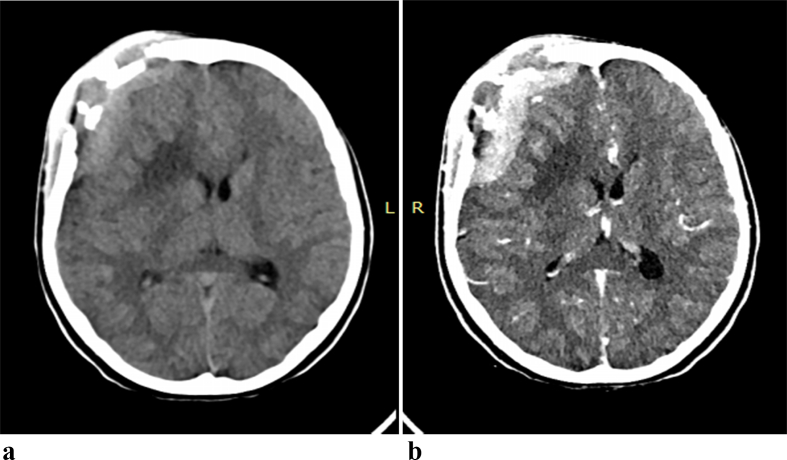
a. Axial non contrast computed tomography showing bony defect, with slightly hyperdense epidural and subgaleal lesion with mixed hypo dense areas plus adjacent brain parenchyma hypo density b. Axial contrast-enhanced computed tomography showing bony defect, with enhancing epidural and sub galeal lesion with non enhancing hypodense areas plus adjacent brain parenchyma hypo density.

CT scan of the head revealed an extensive bony defect involving both the inner and outer tables, with a slightly hyperdense epidural and subgaleal lesion with mixed hypodense areas plus adjacent brain parenchyma hypodensity [Fig. 1, Fig. 2]. The epidural and subgaleal lesion showed enhancement with gadolinium.

**Fig. 3:**
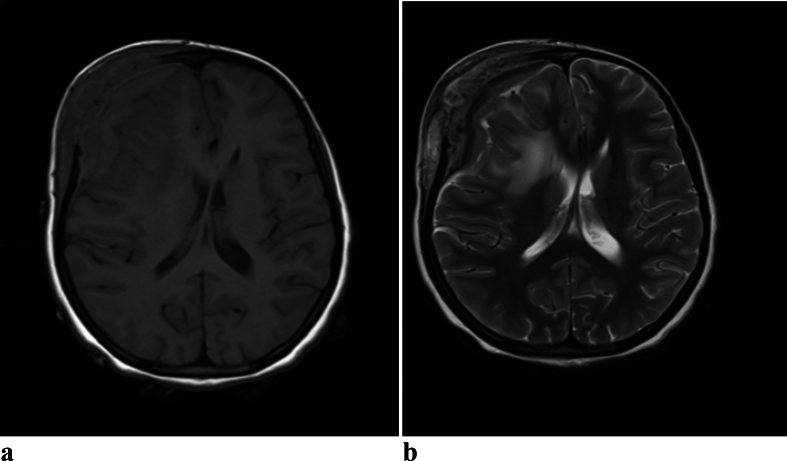
a. Axial T1W1 MRI image of brain showing iso to hyperintense epidural and subgaleal lesion with multiple hypo intense areas having mass effect on the brain and lateral ventricle with midline shift b. Axial T2W2 MRI image of brain showing iso to hyperintense epidural and subgaleal lesion with intervening hypo intense area and hyperintense signal change in the adjacent brain with mass effect on the brain and lateral ventricle with midline shift.

**Fig. 4:**
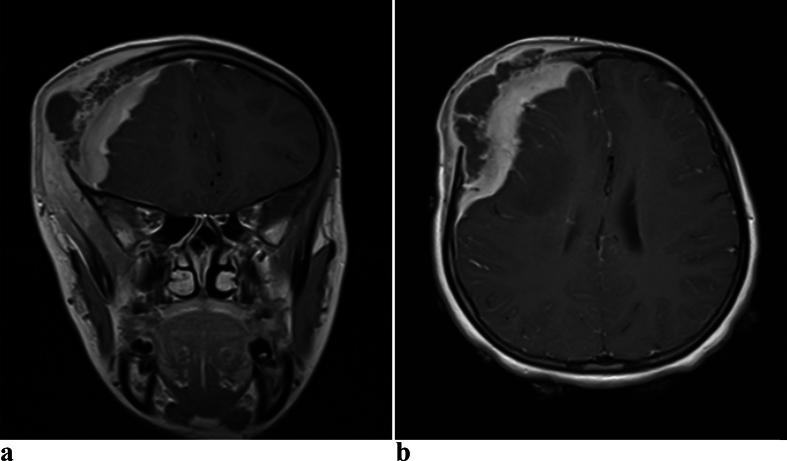
a. Contrast MRI T1W1 coronal image of brain showing extradural and subgaleal enhancing lesion with central mixed enhancing and non enhancing hypo intense area with mass effect and midline shift b. Contrast MRI T1W1 axial image of brain showing extradural and subgaleal enhancing lesion with central non enhancing hypo intense area with mass effect on the brain and lateral ventricle with midline shift.

Brain MRI showed T1 iso to hyperintense and T2 iso to hyperintense epidural and subgaleal lesion with intervening hypointense areas [Fig. 3, Fig. 4]. T2 image also showed hyperintense signal change in the adjacent brain with mass effect on the brain and lateral ventricle with midline shift. The epidural and subgaleal lesion enhanced homogeneously with non-enhancing hypodense areas in between.

**Fig. 5:**
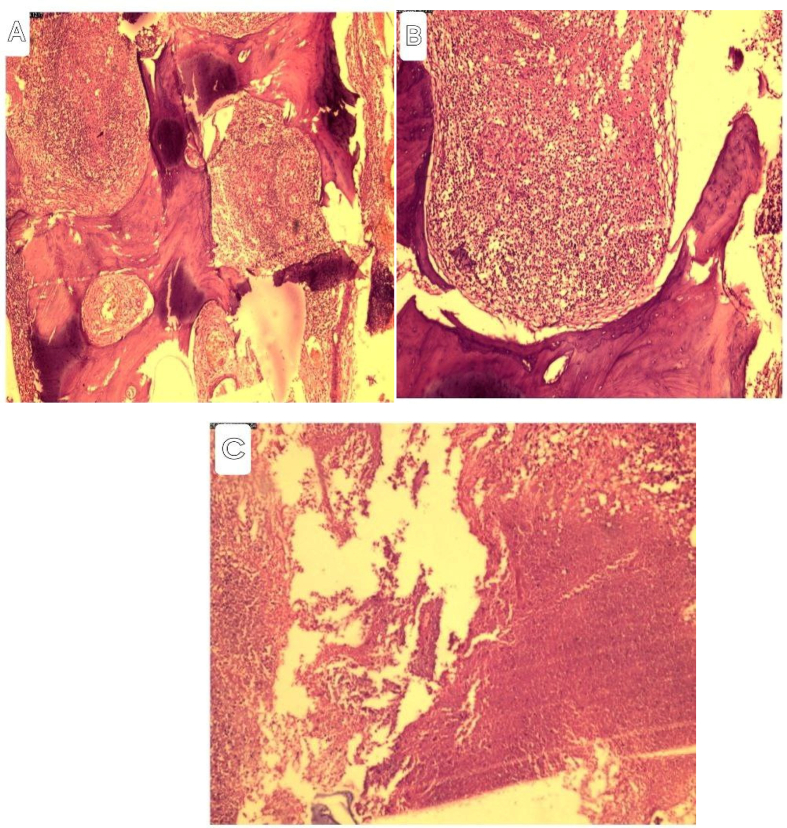
A. Photomicrography of bony tissue with epithelioid granulomas, Low power magnification x100 (A), Well-formed granuloma,(medium power magnification x200) and (C)Areas of extensive necrosis (Magnification x400) Microscopy showed bony tissue fragment with extensive areas of well-formed epithelioid granuloma with central areas of necrosis.

Surgical intervention was performed, revealing extensive destruction of the frontal bone involving both the inner and outer tables. There was a non-offensive thin whitish collection in both the subgaleal and epidural spaces. The epidural space was filled with a soft whitish cheesy collection with underlying granulomatous lesion adherent to the dura. The destroyed bone was removed, and punching of the bone around the lesion was done until healthy bone was found. Thorough debridement of the devitalized tissue was done, and then the space was washed with saline and 2 % hydrogen peroxide. The adherent granulomatous lesion was removed from the dura. There was no extension of the lesion into the subdural spaces. Samples were taken from the bone, subgaleal, and epidural collections, and the skin was closed without replacing the bone. Tissue sent for microscopy showed bony tissue fragments with extensive areas of well-formed epithelioid granulomas with central areas of necrosis [Fig. 5a, b, and c].

After receiving the histopathological examination report, the patient was started on antitubercular therapy with isoniazid: 4–6 mg/kg once daily (200 mg), rifampicin: 8–12 mg/kg once daily (450 mg), pyrazinamide: 20–30 mg/kg once daily (1000 mg), and streptomycin: 15–25 mg/kg once daily (800 mg). She just completed the intensive phase and will continue taking isoniazid and rifampicin for the next 10 months.

The patient's recovery is progressing well. The swelling has subsided, and the wound has healed nicely, with stitches removed on the 12th post-operative day. At the 2nd-month follow-up, the patient returned to our clinic. Except for minimal depression over the craniectomy site, she had significant improvement in headaches and has returned to school and is doing well. We discussed options for cranioplasty and are planning to do it when the school is closed.

## 3 Discussion

Skeletal tuberculosis occurs in approximately 1 % of cases of mycobacterial infection, and calvarial tuberculosis accounts for 0.2 % to 1.3 % of all cases of skeletal tuberculosis. Calvarial tuberculosis predominantly affects the younger population, with approximately 75 % to 90 % of patients being under 20 years of age and 50 % in those under the age of 10 years. [2] This condition is rarely observed in infancy, possibly due to the limited presence of cancellous bone in infants. [5] Furthermore, the disease affects both males and females equally, without any specific sexual preference. [5] Parietal and frontal bones are usually involved due to their high cancellous portion. [1] This corresponds with our patient, where the frontal bone was involved, and her age (17 years) aligns with the predominantly affected age groups.

The spread of the tubercular process to the calvarial bones occurs via the bloodstream. [1] Interestingly, concentrically arranged proliferating fibroblasts encircle the tubercular granulation tissue, acting as a barrier to prevent its extension through the diploe (the spongy layer within the skull). However, if containment fails, the process can extend through either of the tables. [8]

While the primary event in most cases of tuberculosis (TB) involves the infection lodging after hematogenous spread from an extra-calvarial focus, [5] an alternative route via the lymphatic system may be more likely in calvarial tuberculosis. [2] This could explain the rarity of TB in the skull, which is deficient in lymphatics but abundant in vascularity.

Trauma has been implicated as a possible cause of skull tuberculosis. Trauma may lead to increased vascularity, decreased resistance, or the unmasking of latent infections. [2] Additionally, inflammatory cells attracted to the trauma site could potentially harbor intracellular bacilli. However, the role of trauma is probably coincidental rather than causal. [5] In our case, the patient had a history of trauma to the same site of the current lesion 1 year and 6 months ago, and adding the latent characteristics of tuberculosis infection, the trauma could be the possible cause of calvarial tuberculosis in this case.

Early clinical signs are usually absent. However, the emergence of a painless, fluctuant swelling stands out as the most common presentation and typically serves as the initial symptom. [2] This soft swelling of the scalp, accompanied by erosion of the underlying bone, is highly characteristic. The frontal and parietal bones are the most frequently affected sites, whereas involvement of the occipital bone is less common. [4] Notably, clinical findings suggestive of intracranial extension—such as seizures, meningitis, and venous sinus thrombosis—are rarely observed. [5] Our patient presented with a painless fluctuant swelling over the right frontoparietal area with erosion of the underlying bones.

Headache, if present, is usually localized to the site of infection. [2] Rarely, patients may present with seizures or motor deficits [4] Sutures are not a strong barrier to spread, but the dura usually prevents intradural extension. An extensive area of destruction occurs before clinical presentation. Symptoms related to intracranial extension are rare. [4] In our case, as shown in multiple images, the involvement of the adjacent parietal bone illustrated that sutures are not a strong barrier. Despite the mass effect on the ipsilateral lateral ventricle and adjacent brain, there was no intradural extension of the lesion.

Radiological findings, which are very valuable tools to reach a diagnosis, are known to be variable and nonspecific. [5] A CT finding varies from extradural enhancing collection, calvarial destruction, subgaleal collection, sinus formation, and sometimes parenchymal involvement in various combinations (10). MRI is also a valuable imaging modality, and T2-weighted images show a high-signal-intensity soft tissue mass within the defect in the bone. This may project into the subgaleal and/or epidural spaces and show peripheral capsular enhancement on the contrast-enhanced image. [6] The differential diagnoses include pyogenic osteomyelitis, calvarial metastases, multiple myeloma, hemangioma, giant cell tumor, or even an aneurysmal bone cyst and Langerhans cell histiocytosis. [8]

The Mantoux test and elevated erythrocyte sedimentation rate (ESR) serve as valuable markers and can provide diagnostic clues for tuberculosis (TB). However, it is important to note that the Mantoux test may yield negative results in approximately 10 % of patients, and the ESR may remain normal in a similar proportion of cases. [5]

While demonstrating acid-fast bacilli (AFB) on microscopy and obtaining bacterial growth in culture remain the gold standard for diagnosing tuberculosis (TB), these methods may not always be feasible. [4] In such cases, the presence of characteristic granulomas on histology and a positive response to empirical anti-tuberculosis treatment (ATT) serve as evidence of the infection being tubercular. [3]

Treatment for calvarial tuberculosis includes surgery and antituberculous therapy. [9] Although there are some reports in the literature favoring antituberculous therapy alone, recent studies indicate that combination treatment is better, as extensive areas of diseased bone may be a source of tuberculous bacilli unless surgically removed. [4] Surgery is indicated to establish the diagnosis, to remove thick extradural granulation tissue and necrotic bone, and in patients with sinus discharge, intracranial extensions, and large collections of caseating material causing a mass effect. [9] Surgical treatment includes an incision in normal skin with proper care in raising the flap, excision of pus or necrotic tissue, excision of involved bone and extradural granulations. The dura should be left untouched if there is no intradural extension, which is very rare. [4]

## Patient (Parent's) consent

Written informed consent was obtained from the patient Parents for publication of this case report and accompanying images. A copy of the written consent is available for review by the Editor-in-Chief of this journal on request.

## Ethical approval

Ethical approval was provided by the author's institution.

## Source of funding

N/A

## Author contribution

1. Abdulsemed Mohammed Yasin, MD, Assistant professor of Neurosurgery: conceived, wrote, and submitted the report. Involved in the diagnosis, management and follow up of the patient.
2. Eyob Zenebe, MD, Assistant professor of Neurosurgery: Operated on the patient. Reviewed the case report.
3. Kibruyisfaw Zewude, MD, Associate professor of Neurosurgery: Reviewed the case report and Involved in the diagnosis, management and follow up of the patient
4. Dagnachew Tamrat Belete, MD, Assistant Professor of Pathology: Involved in examining the pathology specimen and reviewing the case report
5. Betelhem Gebreamlak, MD, Pathology resident: Involved in examining the pathology specimen

## Guarantor

1. Sadik Jemal, MD, Assistant professor of Neurosurgery: Reviewed the case report.
2. Tadelu Mekonen, MD, Neurosurgery resident: Involved in writing the case report.

## Research registry

N/A

## Conflicts of interest

All authors declare that they have no conflict of interest.
